# Music

**Published:** 1873-01

**Authors:** 


### MUSIC

Hath charms to calm the savage breast; it laid the evil spirit in David's day, and calmed the passions of a raging Saul. It has been said that no man can be thoroughly bad who has a taste for the "concord of sweet sounds." The rat and the serpent have been known to creep from their holes, as if to luxuriate in song. The noble Christian doxology, to the tune of Old Hundred, will thrill a whole congregation, as well as a cow. A very ill-tempered man, who was always beating his beasts, or swearing at them in his terrible passion, was known to have the most unruly oxen in the whole neighborhood; but in course of time it was noticed that his oxen were extremely docile; on being asked for his opinion of the reason of the change, it seems that he had changed his ways, became a Christian, and spent a great part of his time in singing psalms. He noticed that it had a quieting effect on his oxen, that they worked better, pulled more steadily; and when they were not disposed to behave well in the team, and were sulky, he would strike up the long meter doxology, and, said he, "it has a surprising effect on my oxen."

Very recently a snake was observed to follow a funeral procession for a mile or more; when the coffin was lowered, it descended into the grave with it, coiled itself around it, and allowed itself to be killed; it was supposed it followed the music until the grave was reached, and when the music ceased, it made an effort to hide itself under the coffin.

Observant housekeepers may have noticed that whenever an Irish cook or chambermaid is singing, it is an infallible sign of deep anger. If you will only let them alone, pay no attention to what is going on, say not a single word unless absolutely necessary, and then as few as possible, and with quiet courtesy, a very few hours will find a change to the meekness of Moses.

By all means cultivate music in the family; begin early, even when the babe is but six months old, accustom its tender ears to the musical tone, and first of all, to the "songs of the sanctuary," to be remembered pleasantly until the last day of life.

